# Development of somatic mutation signatures for risk stratification and prognosis in lung and colorectal adenocarcinomas

**DOI:** 10.1186/s12920-018-0454-7

**Published:** 2019-01-31

**Authors:** Mark Menor, Yong Zhu, Yu Wang, Jicai Zhang, Bin Jiang, Youping Deng

**Affiliations:** 1 Department of Complementary & Integrative Medicine, University of Hawaii John A. Burns School of Medicine, Honolulu, HI USA; 2 National Medical Centre of Colorectal Disease, The Third Affiliated Hospital of Nanjing University of Chinese Medicine, Nanjing, People’s Republic of China; 3 Department of Oncology, The Third Affiliated Hospital of Nanjing University of Chinese Medicine, Nanjing, 210001 Jiangsu Province China; 4 Department of Laboratory Medicine, Shiyan Taihe Hospital, College of Biomedical Engineering, Hubei University of Medicine, Shiyan, Hubei 442000 People’s Republic of China

**Keywords:** TGCA, Somatic mutation, Prognosis, Lung adenocarcinoma, Colorectal adenocarcinoma

## Abstract

**Background:**

Prognostic signatures are vital to precision medicine. However, development of somatic mutation prognostic signatures for cancers remains a challenge. In this study we developed a novel method for discovering somatic mutation based prognostic signatures.

**Results:**

Somatic mutation and clinical data for lung adenocarcinoma (LUAD) and colorectal adenocarcinoma (COAD) from The Cancer Genome Atlas (TCGA) were randomly divided into training (*n* = 328 for LUAD and 286 for COAD) and validation (*n* = 167 for LUAD and 141 for COAD) datasets. A novel method of using the log2 ratio of the tumor mutation frequency to the paired normal mutation frequency is computed for each patient and missense mutation. The missense mutation ratios were mean aggregated into gene-level somatic mutation profiles. The somatic mutations were assessed using univariate Cox analysis on the LUAD and COAD training sets separately. Stepwise multivariate Cox analysis resulted in a final gene prognostic signature for LUAD and COAD. Performance was compared to gene prognostic signatures generated using the same pipeline but with different somatic mutation profile representations based on tumor mutation frequency, binary calls, and gene-gene network normalization. Signature high-risk LUAD and COAD cases had worse overall survival compared to the signature low-risk cases in the validation set (log-rank test *p*-value = 0.0101 for LUAD and 0.0314 for COAD) using mutation tumor frequency ratio (MFR) profiles, while all other methods, including gene-gene network normalization, have statistically insignificant stratification (log-rank test p-value ≥0.05). Most of the genes in the final gene signatures using MFR profiles are cancer-related based on network and literature analysis.

**Conclusions:**

We demonstrated the robustness of MFR profiles and its potential to be a powerful prognostic tool in cancer. The results are robust according to validation testing and the selected genes are biologically relevant.

## Background

Lung and colon cancer are the leading cause of death over all cancers in the United States in 2017, with 155,870 and 50,260 deaths, respectively [1]. Prognostic signatures and risk stratification are vital to clinical decision making of treatment options in cancer precision medicine. As patient prognosis remains poor [2], researchers are seeking to develop improved prognostic signatures using molecular information, such as incorporating long non-coding RNA expression [3, 4].

However, incorporating somatic mutation profiles into prognostic signatures has remained a challenge and is often overlooked due to the sparse and binary nature of somatic mutation data [5]. The sparsity of the data arises from the observation that the vast majority of mutated genes are not shared among patients [6]. Save for a few frequently mutated driver genes, most somatically mutated genes are likely to be composed of only passenger mutations that do not provide growth advantage [7].

To investigate the prognostic value of somatic mutations, studies have chosen to tackle the challenge by confronting the sparsity problem. Le Morvan et al. [8] uses gene-gene networks as prior knowledge to de-sparsify the data. A patient’s binary somatic mutation profile is transformed by removing non-essential mutations and adding proxy mutations based on gene-gene network topology to normalize tumor mutational burden within a sample of patients. However, gene-gene networks vary from tissue to tissue and a single set of canonical gene-gene networks as prior knowledge may omit or overemphasize some interactions [9]. To address this issue, other studies have elected to use cancer-specific co-expression networks based on RNA expression data [10] or canonical pathways [11].

In this study, we confront the challenge of the binary nature of somatic mutation data rather than the sparsity problem. We propose the usage of the quantitative mutation frequency ratio of tumor vs. normal tissue from whole exome sequencing in building somatic mutation profiles. Using somatic mutation data for lung adenocarcinoma (LUAD) and colorectal adenocarcinoma (COAD) from The Cancer Genome Atlas (TCGA) [12, 13], we evaluate the risk stratification and prognostic performance of somatic mutation signatures generated by using two types of continuous somatic mutation profiles: mutation frequency ratio (MFR) profiles and tumor mutation frequency (TMF) profiles. We compare to two existing types of binary mutation profiles, raw binary mutation (BM) profiles and gene-gene network normalized profiles provided by NetNorM [8]. We show that the somatic mutation signatures generated by MFR profiles consistently provides statistically significant risk stratification while the other types of profiles do not.

## Results

### Identification of prognostic somatically mutated genes

**Fig. 1:**
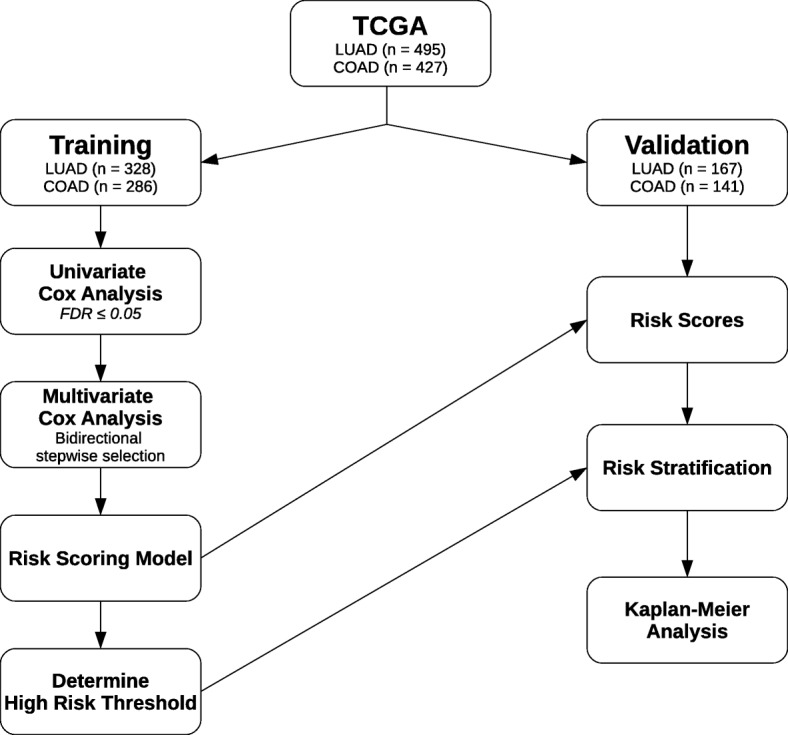
Identification of prognostic somatic mutation gene signature. DNA-seq prognostic analysis and signature generation pipeline. TCGA somatic mutation data is randomly split into training and validation datasets. Univariate Cox analysis identifies mutated genes associated with survival and only significant genes (FDR ≤ 0.05) are considered further. Bidirectional stepwise model selection for multivariate Cox analysis is used to select the final prognostic somatic mutation gene signature. Risk scores for patients in both training and datasets are computed using the final signature. The 75% percentile risk score of the training dataset is used as the stratification threshold for the KM analysis on both the training and validation datasets

To identify and evaluate prognostic somatically mutated genes using different types of somatic mutation profiles, we used a pipeline (Fig. 1) adapted from Shukla et al.’s RNA-seq pipeline [3]. Clinical and controlled somatic mutation data for LUAD and COAD was gathered from TCGA [12, 13]. The data (Table 1) was partitioned randomly into training (*n* = 328 for LUAD and *n* = 286 for COAD) and validation (*n* = 167 for LUAD and *n* = 141 COAD) datasets and somatic mutation profiles generated.

**Table 1:** Clinical characteristics of the patients

Factor	TCGA LUAD Training	TCGA LUAD Validation	TCGA COAD Training	TCGA COAD Validation
Num. of patients	328	167	286	141
Age, years, mean (SD)	65.8 (10.2)	64.5 (9.6)	66.6 (12.8)	66.4 (13.5)
Median survivor follow-up, days	506.5	218.0	716.5	730.0
Female, num. (%)	169 (51.5)	97 (58.1)	151 (52.8)	52 (36.9)
Stage I, num. (%)	176 (53.7)	90 (54.0)	50 (17.5)	22 (15.6)
Stage II, num. (%)	77 (23.5)	39 (23.4)	101 (35.3)	62 (44.0)
Stage III, num. (%)	49 (14.9)	31 (18.6)	75 (26.2)	44 (31.2)
Stage IV, num. (%)	21 (6.4)	5 (3.0)	50 (17.5)	12 (8.5)

Four different types of somatic mutation profiles were considered: MFR, TMF, BM, and NetNorM profiles. The somatic mutation profile of a single patient is a vector with an element for every gene. The BM profile of a patient consists of a sparse binary vector where an element denotes if a gene is somatically mutated or not. The NetNorM profile was generated from the BM profile by normalizing the number of mutated genes via the removal or addition of somatically mutated genes [8]. While the NetNorM profile remains binary in nature, its process mitigates the sparsity problem of somatic mutation data by incorporating gene-gene network prior knowledge.

Additionally, we propose the usage of MFR and TMF profiles, which to the best of our knowledge, has not be considered previously in the literature to confront the difficulties of working with sparse binary data. TMF profiles incorporate the tumor data on the number of reads supporting the mutation vs. the reference genome. The MFR takes it a step further and considers the mutation frequency ratio of the tumor sample vs. the paired normal tissue sample. Both TMF and MFR profiles use continuous rather than binary values for somatic mutation profile representation.

Individually for each type of somatic mutation profile and tumor type, somatic mutation based prognostic signatures are generated using the pipeline outlined in Fig. 1. Univariate Cox proportional hazards regression is first performed on the training dataset to short list prospective genes with a FDR cutoff of 0.05. The prospective genes are then subjected to bidirectional stepwise multivariate Cox proportional hazards regression model selection to the determine the final prognostic signature (Table 2 and Table 3). We verified that all of the final prognostic signatures do not violate the proportional hazards assumption using the Schoenfeld Residual Test.

**Table 2:** Genes found in prognostic somatic mutation gene signatures for LUAD

Gene Symbol	MFR	TMF	BMF	NetNorM
ABCB6	TRUE	FALSE	TRUE	FALSE
MSANTD3	TRUE	FALSE	FALSE	FALSE
CFAP69	TRUE	FALSE	FALSE	FALSE
CHST5	TRUE	FALSE	FALSE	FALSE
ZNF768	TRUE	FALSE	FALSE	FALSE
NDN	TRUE	FALSE	FALSE	FALSE
SERPINI2	TRUE	FALSE	FALSE	FALSE
FGD3	TRUE	TRUE	TRUE	TRUE
SLC29A4	TRUE	TRUE	TRUE	FALSE
HSD17B4	TRUE	TRUE	TRUE	FALSE
OR5H15	TRUE	FALSE	TRUE	FALSE
PFKM	TRUE	FALSE	FALSE	FALSE
MADD	TRUE	FALSE	FALSE	FALSE
PODN	TRUE	FALSE	TRUE	FALSE
MMP8	TRUE	TRUE	FALSE	FALSE
ARHGAP4	TRUE	FALSE	FALSE	FALSE
SDHA	TRUE	TRUE	TRUE	FALSE
C3orf20	TRUE	FALSE	FALSE	FALSE
HEATR1	TRUE	FALSE	FALSE	FALSE
MYOT	TRUE	FALSE	FALSE	FALSE
AOC1	FALSE	TRUE	TRUE	FALSE
TLR9	FALSE	TRUE	FALSE	FALSE
MOSPD2	FALSE	TRUE	TRUE	TRUE
EPHA2	FALSE	TRUE	TRUE	TRUE
ZNF880	FALSE	TRUE	FALSE	FALSE
TAS2R39	FALSE	TRUE	FALSE	FALSE
DNTTIP1	FALSE	TRUE	FALSE	FALSE
HHAT	FALSE	TRUE	TRUE	FALSE
ALOXE3	FALSE	TRUE	TRUE	FALSE
PRMT5	FALSE	TRUE	TRUE	FALSE
FAM83B	FALSE	TRUE	FALSE	FALSE
BEST4	FALSE	TRUE	FALSE	FALSE
BCAS3	FALSE	TRUE	FALSE	FALSE
MAP3K1	FALSE	TRUE	FALSE	FALSE
GPR52	FALSE	TRUE	FALSE	FALSE
DNAJC10	FALSE	TRUE	FALSE	FALSE
ADGRG7	FALSE	TRUE	FALSE	FALSE
CDRT15	FALSE	TRUE	FALSE	FALSE
MOCS3	FALSE	TRUE	FALSE	FALSE
C5	FALSE	TRUE	FALSE	FALSE
CNTN1	FALSE	TRUE	FALSE	FALSE
CLCN2	FALSE	TRUE	FALSE	FALSE
CBLB	FALSE	TRUE	TRUE	TRUE
MSH3	FALSE	TRUE	FALSE	FALSE
RBM45	FALSE	TRUE	FALSE	FALSE
SQRDL	FALSE	FALSE	TRUE	FALSE
LIPE	FALSE	FALSE	TRUE	FALSE
TBPL2	FALSE	FALSE	TRUE	FALSE
LANCL2	FALSE	FALSE	TRUE	FALSE
BMP6	FALSE	FALSE	TRUE	FALSE
TTLL4	FALSE	FALSE	TRUE	FALSE
NPAS1	FALSE	FALSE	TRUE	FALSE
ALX4	FALSE	FALSE	TRUE	FALSE
CRNN	FALSE	FALSE	TRUE	FALSE
LRRC4	FALSE	FALSE	TRUE	FALSE
NPC1L1	FALSE	FALSE	TRUE	TRUE
TYRO3	FALSE	FALSE	FALSE	TRUE
TOP2A	FALSE	FALSE	FALSE	TRUE
SIGLEC10	FALSE	FALSE	FALSE	TRUE
AQP6	FALSE	FALSE	FALSE	TRUE
ZC3H7B	FALSE	FALSE	FALSE	TRUE
IGHG2	FALSE	FALSE	FALSE	TRUE
TTI1	FALSE	FALSE	FALSE	TRUE
MEGF10	FALSE	FALSE	FALSE	TRUE
TRIM8	FALSE	FALSE	FALSE	TRUE
ZNF714	FALSE	FALSE	FALSE	TRUE
FOXO4	FALSE	FALSE	FALSE	TRUE
OR3A1	FALSE	FALSE	FALSE	TRUE
COL24A1	FALSE	FALSE	FALSE	TRUE
COPE	FALSE	FALSE	FALSE	TRUE
PCDH7	FALSE	FALSE	FALSE	TRUE
SLC25A24	FALSE	FALSE	FALSE	TRUE
FUT9	FALSE	FALSE	FALSE	TRUE
MAGI2	FALSE	FALSE	FALSE	TRUE
ZNF148	FALSE	FALSE	FALSE	TRUE
BAZ2B	FALSE	FALSE	FALSE	TRUE

List of somatically mutated genes selected by the pipeline for LUAD using each type of somatic mutation profiles

**Table 3:** Genes found in prognostic somatic mutation gene signatures for COAD

Gene Symbol	MFR	TMF	BMF	NetNorM
ABCB5	FALSE	FALSE	FALSE	TRUE
ACSM5	FALSE	FALSE	FALSE	TRUE
ARHGAP15	TRUE	FALSE	FALSE	FALSE
C11orf53	TRUE	FALSE	FALSE	FALSE
C8B	FALSE	FALSE	TRUE	TRUE
CAPN9	FALSE	TRUE	FALSE	FALSE
CARD11	FALSE	FALSE	FALSE	TRUE
CDH24	TRUE	FALSE	TRUE	FALSE
CER1	TRUE	TRUE	TRUE	FALSE
CHI3L1	TRUE	FALSE	FALSE	FALSE
COG7	TRUE	FALSE	FALSE	FALSE
COL4A4	FALSE	TRUE	FALSE	FALSE
COL9A1	FALSE	FALSE	FALSE	TRUE
CTGLF11P	FALSE	TRUE	FALSE	FALSE
DCAF12	FALSE	TRUE	FALSE	FALSE
DGKB	FALSE	FALSE	FALSE	TRUE
DMKN	TRUE	FALSE	TRUE	FALSE
DNALI1	TRUE	FALSE	TRUE	FALSE
DOCK3	FALSE	FALSE	FALSE	TRUE
EIF3F	FALSE	FALSE	FALSE	TRUE
FBXO38	TRUE	FALSE	FALSE	FALSE
FOXD4L6	FALSE	TRUE	FALSE	FALSE
FSHR	FALSE	TRUE	FALSE	FALSE
GRPR	FALSE	TRUE	FALSE	FALSE
H2AFY2	FALSE	FALSE	TRUE	FALSE
HIF1AN	FALSE	TRUE	FALSE	FALSE
IGHA1	TRUE	FALSE	FALSE	FALSE
IQCH	TRUE	FALSE	FALSE	FALSE
KANSL3	TRUE	TRUE	FALSE	FALSE
KRT73	FALSE	FALSE	FALSE	TRUE
MARCH11	TRUE	FALSE	TRUE	FALSE
MEOX1	TRUE	FALSE	FALSE	FALSE
METTL21C	TRUE	FALSE	TRUE	TRUE
MICA	TRUE	TRUE	TRUE	FALSE
NAV1	FALSE	FALSE	TRUE	FALSE
NKD1	TRUE	TRUE	TRUE	FALSE
NTSR1	FALSE	TRUE	FALSE	FALSE
OGFR	FALSE	FALSE	FALSE	TRUE
OR10A7	FALSE	TRUE	FALSE	FALSE
OR10H2	FALSE	FALSE	FALSE	TRUE
OR11H1	FALSE	FALSE	FALSE	TRUE
OR13C8	FALSE	TRUE	FALSE	FALSE
OR1D5	FALSE	FALSE	TRUE	TRUE
PDHB	TRUE	FALSE	FALSE	FALSE
PDPR	FALSE	FALSE	FALSE	TRUE
PRKG2	TRUE	FALSE	FALSE	FALSE
PSMD2	TRUE	FALSE	FALSE	FALSE
RANBP17	TRUE	FALSE	FALSE	TRUE
RARG	FALSE	TRUE	FALSE	FALSE
RBM22	FALSE	FALSE	FALSE	TRUE
RERG	TRUE	FALSE	TRUE	FALSE
RP11.231C14.4	TRUE	FALSE	FALSE	FALSE
SAGE1	FALSE	TRUE	FALSE	FALSE
SCD5	FALSE	TRUE	FALSE	FALSE
SDR9C7	TRUE	FALSE	FALSE	FALSE
SERPINB3	TRUE	TRUE	FALSE	FALSE
SPDYE5	FALSE	TRUE	FALSE	FALSE
SUSD2	FALSE	FALSE	FALSE	TRUE
TREH	FALSE	FALSE	FALSE	TRUE
UBL4B	FALSE	TRUE	FALSE	FALSE
UBTD1	TRUE	FALSE	FALSE	FALSE
UBTFL1	TRUE	FALSE	FALSE	FALSE
USP50	TRUE	FALSE	FALSE	FALSE
VPS36	FALSE	FALSE	FALSE	TRUE
WDR7	FALSE	FALSE	FALSE	TRUE
ZNF133	TRUE	TRUE	FALSE	FALSE
ZNF214	TRUE	TRUE	TRUE	FALSE
ZNF586	TRUE	FALSE	FALSE	FALSE
ZNF83	TRUE	FALSE	FALSE	FALSE

List of somatically mutated genes selected by the pipeline for COAD using each type of somatic mutation profiles

### Comparison of risk stratification

Kaplan-Meier (KM) survival curves are used to assess and compare the different types of somatic mutation profiles in both the training and validation datasets. Using the final Cox model for risk scoring, the high-risk threshold for stratification in both the training and validation datasets was chosen to be the 75th percentile of the risk scores in the training dataset.

**Fig. 2:**
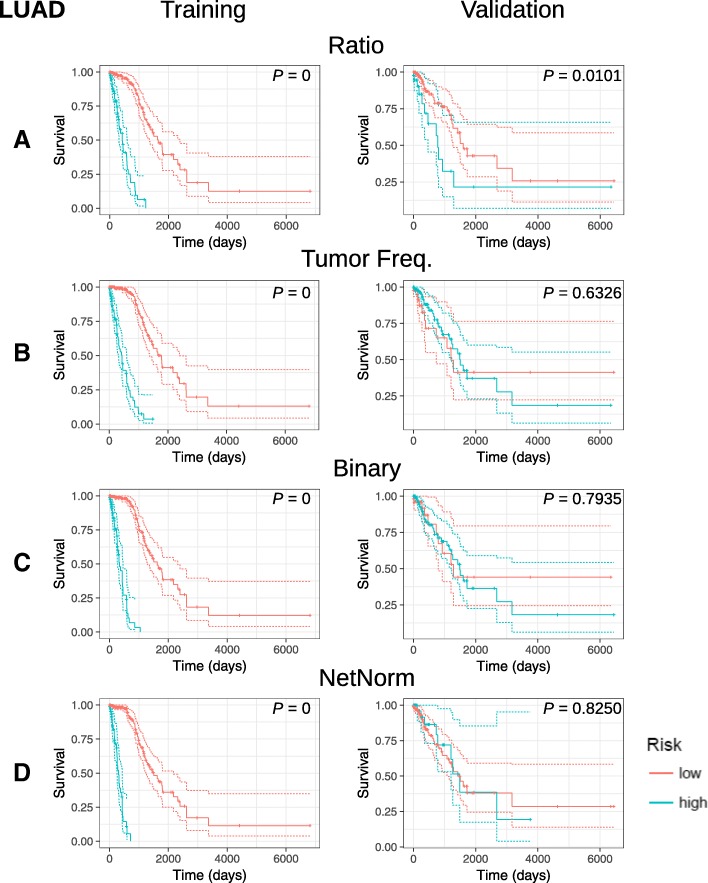
Kaplan-Meier analysis of prognostic somatic mutation gene signatures. KM survival curves for LUAD training and validation datasets using (**a**) MFR, (**b**) TMF, (**c**) BM, and (**d**) NetNorM somatic mutation profiles

**Fig. 3:**
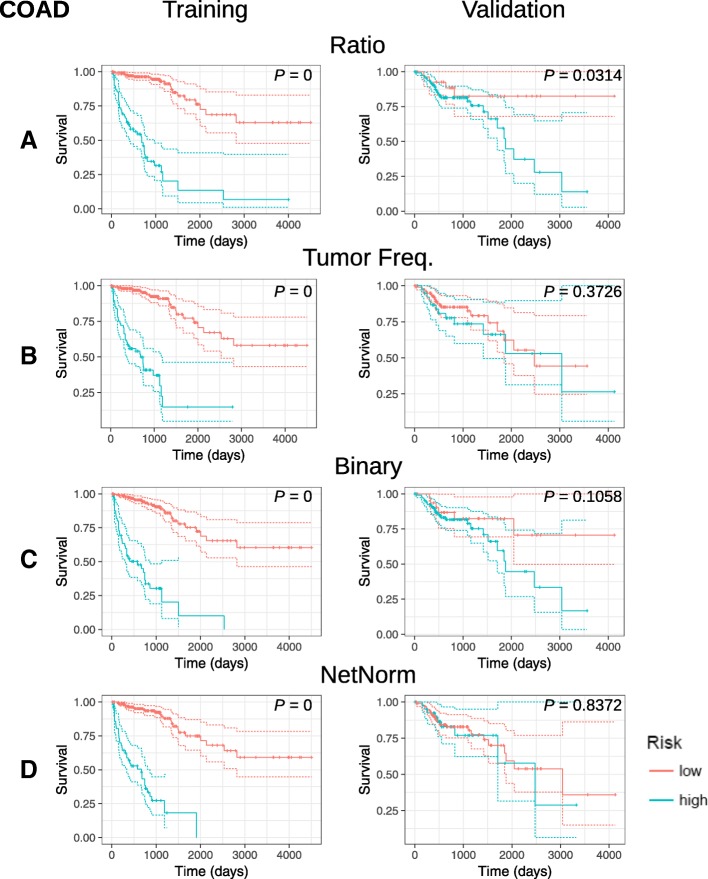
Kaplan-Meier analysis of prognostic somatic mutation gene signatures. KM survival curves for COAD training and validation datasets using (**a**) MFR, (**b**) TMF, (**c**) BM, and (**d**) NetNorM somatic mutation profiles

**Fig. 4:**
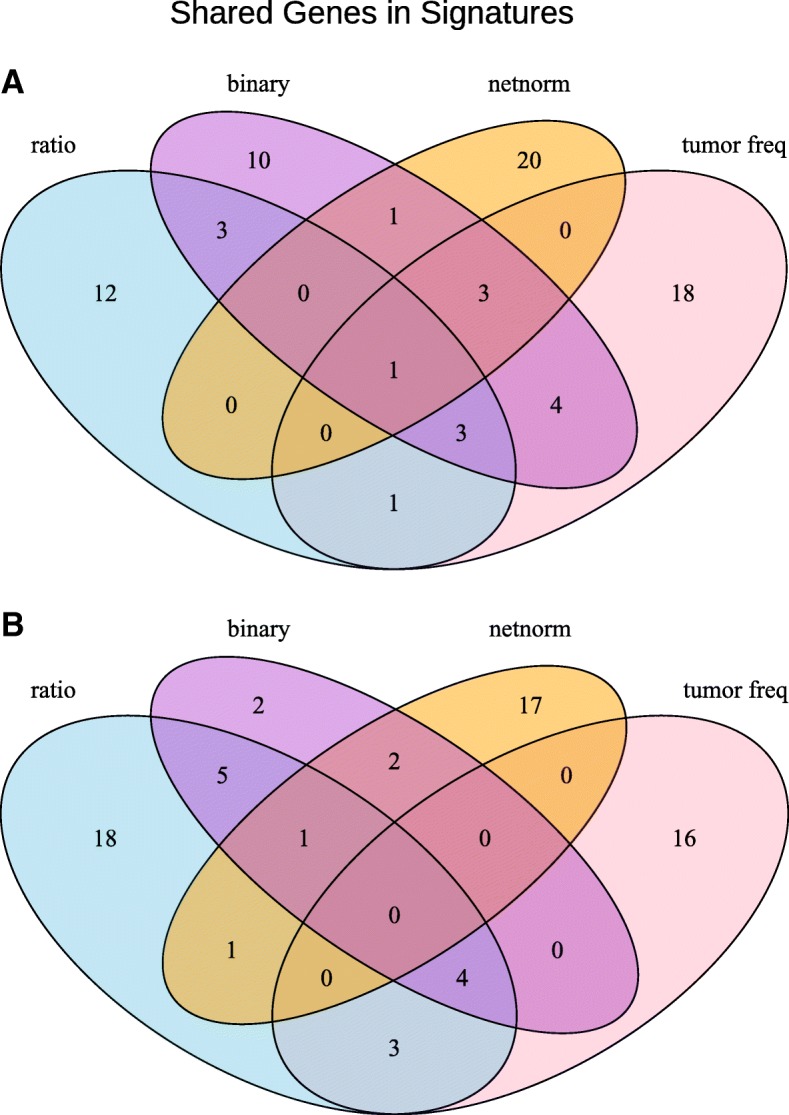
Selected somatically mutated genes for signatures. Venn diagrams of selected genes using MFR, TMF, BM, and NetNorM somatic mutation profiles for (**a**) LUAD and (**b**) COAD

We observed that all somatic mutation profile types achieve significant risk stratification on the training dataset (log rank test *p*-value ≈ 0) for both LUAD and COAD (Fig. 2, Fig. 3). For both LUAD and COAD, however, only the stratification generated by MFR profiles is statistically significant in the validation datasets (log rank test p-value = 0.0101 for LUAD, 0.0314 for COAD) (Fig. 2a, Fig 3a), while all other profiles, including NetNorM, are not statistically significant (Fig. 2b, c and d, Fig. 3b, c and d). Furthermore, the final prognostic signatures generated by each type of somatic mutation profile only minimally overlap for both LUAD and COAD cases (Fig. 4).

The results suggest that the MFR profile’s prognostic signature is more robust, while the other types of profiles are subject to harsh overfitting that is typical in contexts with a larger number of covariates than samples. This is consistent with the observation that NetNorM profiles typically do not perform statistically different from binary profiles [8]. De-sparsifying somatic mutation data using gene-gene network prior information does not necessarily lead to improved prognostic and risk stratification performance.

### Somatic mutation gene signatures

**Fig. 5:**
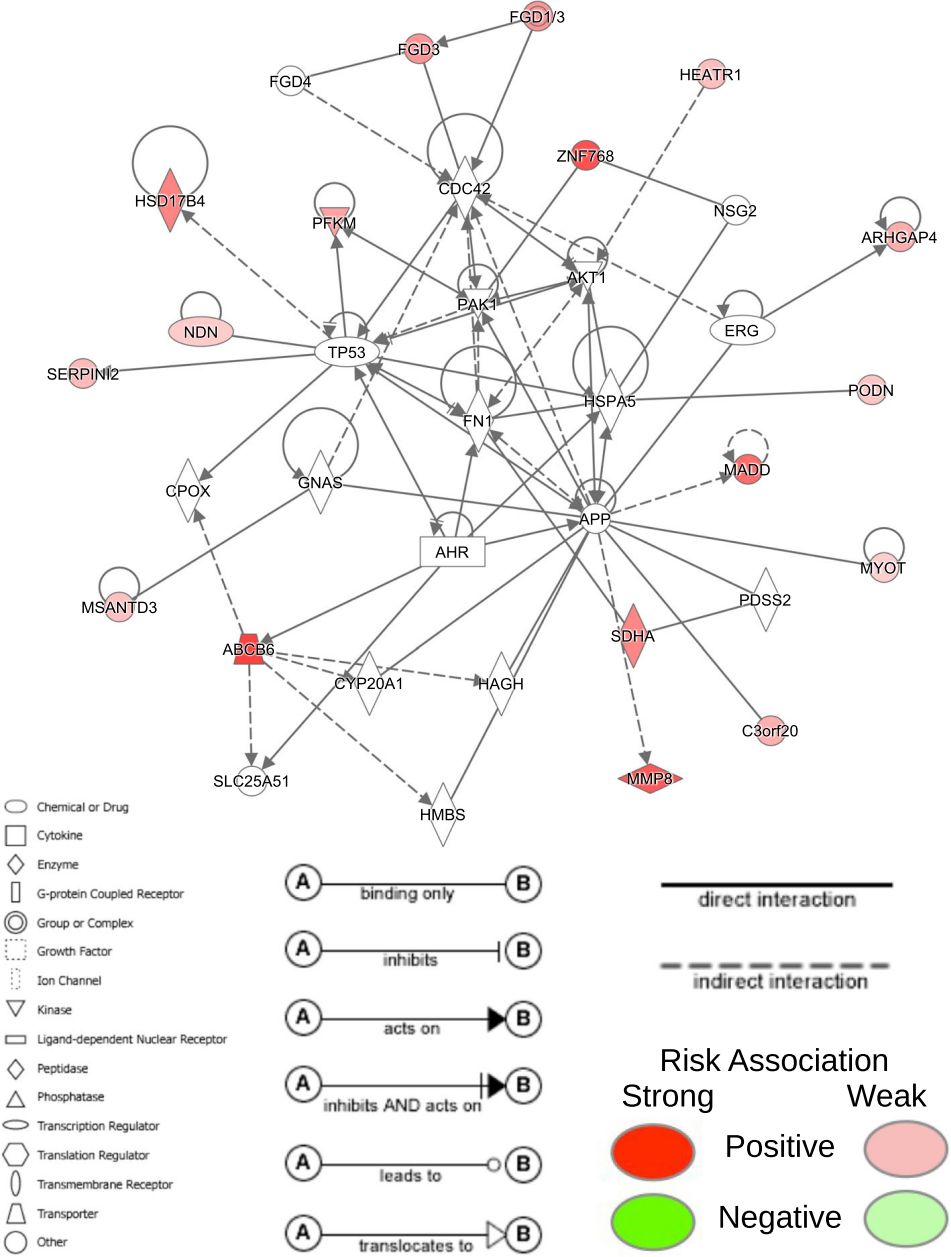
Network for somatically mutated genes in LUAD signature. Network containing 16 of the 20 genes in the LUAD prognostic signature using MFR somatic mutation profiles. The network is associated with cell death and survival, and cellular movement. Red nodes represent genes in the final prognostic signature and denote positive association with risk

A PubMed search of the individual genes and a network analysis of the full signatures using Ingenuity Pathway Analysis (QIAGEN Inc., https://www.qiagenbioinformatics.com/products/ingenuity-pathway-analysis/, accessed: Feb. 14, 2018) was performed to assess the biological relevancy of the final prognostic gene signatures generated by MFR profiles. A network containing 16 of the 20 genes in the LUAD prognostic signature (Table 4) was found (Fig. 5). The network is associated with cell death and survival, and cellular movement. All genes in the prognostic signature are positively associated with risk (denoted in red in Fig. 5). *SDHA* is the gene with the largest coefficient in the risk model (hazard ratio (HR) = 1.877). *SDHA* is a tumor suppressor and is implicated in paraganglioma and gastrointestinal stromal tumors [14]. While association of *SDHA* copy number variation to prognosis was found in lung squamous cell carcinoma [15], we have found no literature exploring the connection of *SDHA* to lung adenocarcinoma.

**Table 4:** Prognostic somatic mutation gene signature for LUAD using MFR profiles

Gene	HR	Lower .95	Upper .95
ABCB6	1.533	1.3460	1.745
MSANTD3	1.154	1.0075	1.321
CFAP69	1.036	0.7275	1.475
CHST5	1.610	1.4081	1.841
ZNF768	1.593	1.3626	1.861
NDN	1.112	0.9857	1.254
SERPINI2	1.187	1.0289	1.369
FGD3	1.379	1.1587	1.642
SLC29A4	1.295	1.1428	1.468
HSD17B4	1.350	1.1723	1.556
OR5H15	1.459	1.2308	1.731
PFKM	1.406	1.1341	1.742
MADD	1.256	1.1484	1.374
PODN	1.153	0.9972	1.332
MMP8	1.396	1.2429	1.569
ARHGAP4	1.421	1.1078	1.822
SDHA	1.877	1.3877	2.538
C3orf20	1.187	1.0468	1.347
HEATR1	1.132	1.0123	1.266
MYOT	1.179	0.9694	1.433

Four additional genes in the LUAD signature also have known associations with lung cancer. *PFKM* has mutations associated with survival outcomes in lung squamous cell carcinoma [16]. *MADD* promotes survival of LUAD cells and is a potential therapeutic target [17]. *SERPINI2* is tumor suppressor gene and is associated with squamous cell lung cancer [18]. Finally, it has been found that certain *MMP8* mutations are correlated with risk of developing lung cancer [19].

Eight of the remaining genes in the LUAD signature are associated with other cancer types and their connection to LUAD is yet uncharacterized. *ABCB6* [20, 21], *ZNF768* [22], and the *TP53*-mediated tumor suppressor gene *NDN* [23] are all associated with colorectal cancers. *MSANTD3* is an oncogene in salivary gland acinic cell carcinoma [24]. *FGD3* is implicated in breast cancer [25] and *ARHGAP4* in ovarian tumors [26]. It has been observed that increased expression of *HSD17B4* is correlated with poor prognosis in prostate cancer [27]. Lastly, correlation of *HEATR1* with shorter overall survival has been shown in pancreatic ductal adenocarcinoma [28].

**Fig. 6:**
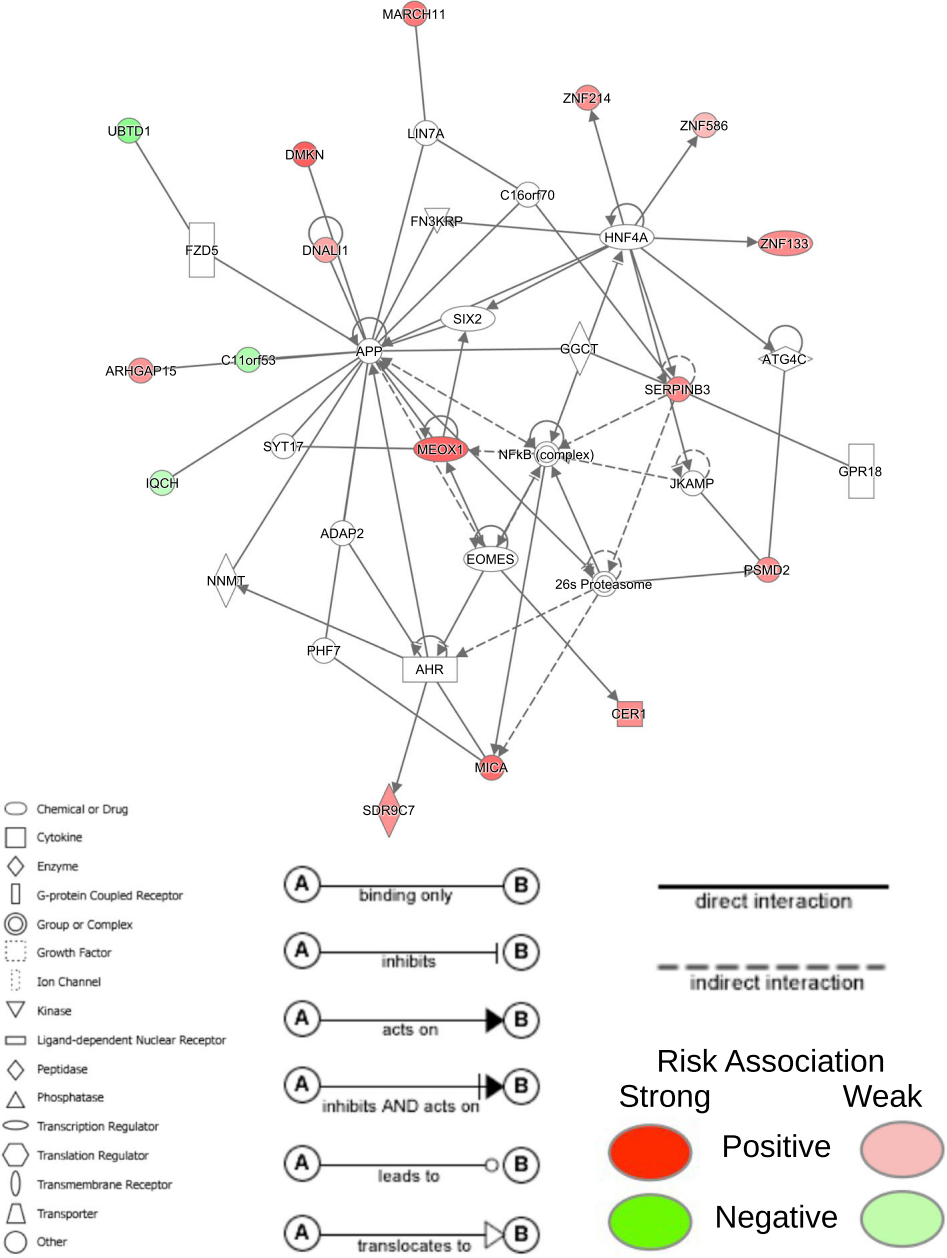
First network for somatically mutated genes in COAD signature Network containing 16 of the 32 genes in the COAD prognostic signature using MFR somatic mutation profiles. The network is associated with embryonic, organismal, and tissue development. Red and green nodes represent genes in the final prognostic signature and denote positive and negative association with risk, respectively

**Fig. 7:**
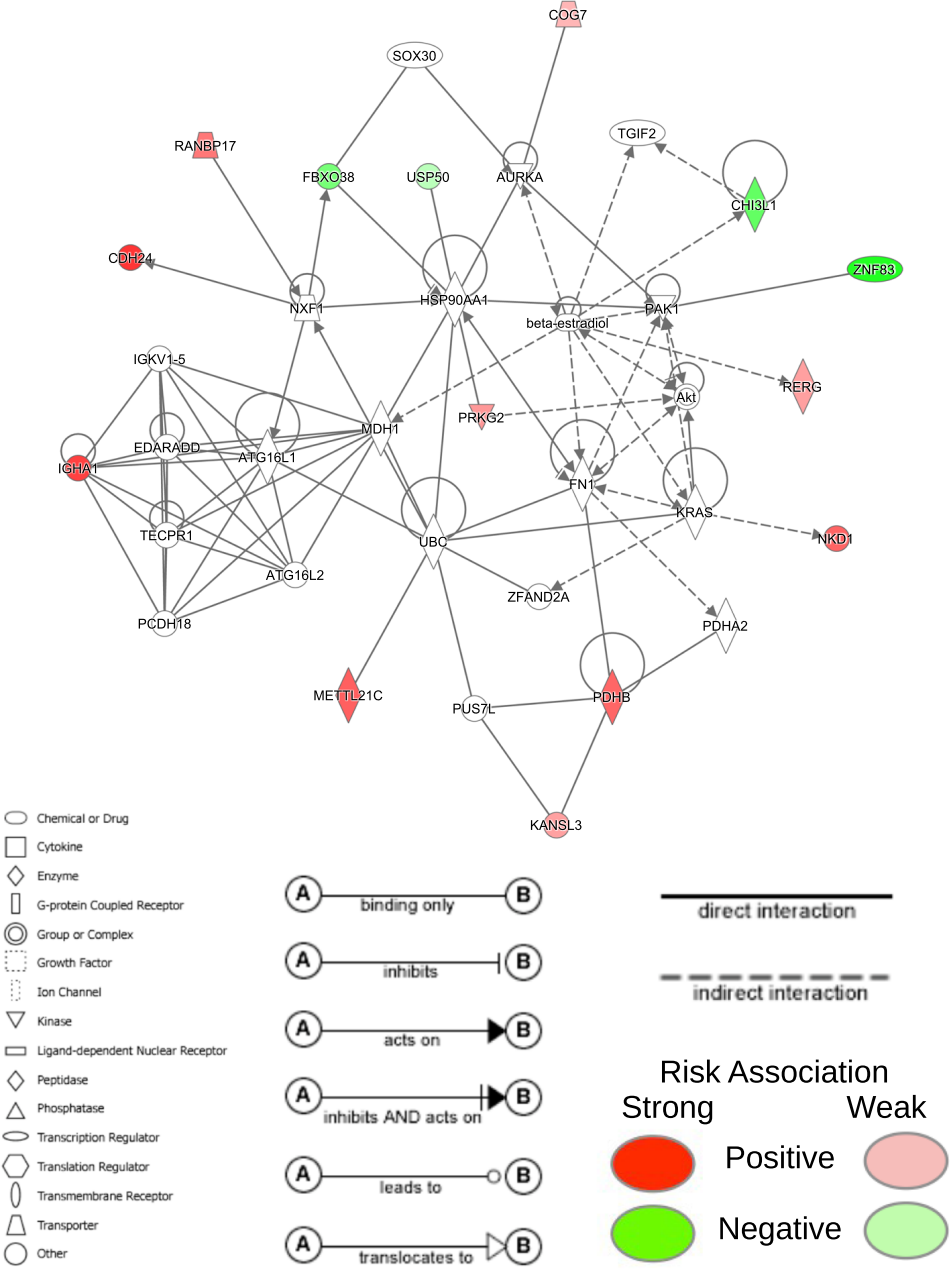
Second network for somatically mutated genes in COAD signature Network containing 14 of the 32 genes in the COAD prognostic signature using MFR somatic mutation profiles. The network is associated with cancer and organismal injury and abnormalities. Red and green nodes represent genes in the final prognostic signature and denote positive and negative association with risk, respectively

For the COAD prognostic signature (Table 5), we found that 30 of the 32 genes were involved in two different networks. The first network contains 16 of the 32 genes in the COAD prognostic signature (Fig. 6) and is associated with embryonic, organismal, and tissue development. The second network contains 14 of the 32 genes in the COAD prognostic signature (Fig. 7) and is associated with cancer and organismal injury and abnormalities. Unlike the LUAD signature where all genes were positively associated with increased risk, mutations in seven of the genes are associated with reduced risk (*USP50*, *UBTD1*, *ZNF83*, *FBX038*, *C11orf53*, *IQCH*, and *CHI3L1*) and are denoted in green in Figs. 6 and 7.

**Table 5:** Prognostic somatic mutation gene signature for COAD using MFR profiles

Gene	HR	Lower .95	Upper .95
DNALI1	1.5329	1.1595	2.0266
CDH24	1.8902	1.5805	2.2607
MICA	1.8827	1.4679	2.4147
METTL21C	1.4121	1.2469	1.5993
IGHA1	1.8858	1.5562	2.2851
UBTFL1	2.3007	1.7595	3.0083
PSMD2	1.3216	1.1431	1.5280
CER1	1.3071	1.1396	1.4994
RERG	1.9545	1.3025	2.9327
ZNF214	1.5077	1.2189	1.8650
MARCH11	1.4303	1.2257	1.6689
USP50	0.7640	0.5805	1.0056
NKD1	1.8210	1.4579	2.2744
UBTD1	0.4835	0.3106	0.7526
MEOX1	1.4101	1.2415	1.6017
KANSL3	1.2496	1.0896	1.4330
ARHGAP15	1.2390	1.1033	1.3913
SERPINB3	1.3768	1.1808	1.6053
ZNF83	0.4153	0.3169	0.5443
DMKN	1.4173	1.2479	1.6097
RP11.231C14.4	3.3855	2.3763	4.8232
SDR9C7	1.3940	1.1702	1.6607
PRKG2	1.2619	1.1085	1.4365
RANBP17	1.2959	1.1605	1.4471
COG7	1.1759	1.0345	1.3367
FBXO38	0.6475	0.5196	0.8068
PDHB	1.8935	1.4885	2.4086
ZNF133	1.4302	1.1948	1.7119
C11orf53	0.7342	0.5643	0.9551
IQCH	0.8654	0.7385	1.0141
CHI3L1	0.2479	0.1338	0.4591
ZNF586	1.2146	1.0322	1.4291

Ten of the genes in the COAD signature are implicated in colorectal cancers (CRC). *MICA* has high cell-surface expression in cancers of the digestive system and have been found to be correlated with increased survival [29]. Copy number variation of *RERG* is correlated with CRC risk [30]. *NKD1* is involved in Wnt signaling central to tumor cell growth in CRC and other cancers [31]. Lower expression of *UBTD1* correlates with worse prognosis [32]. *SERPINB3* has a driving role in more aggressive cellular phenotypes of CRC [33]. *DMKN* has been previously proposed as a biomarker of early-stage CRC [34]. *PDHB* diminishes the oncogenic effects of *miR-146b-5p* on the growth and invasion of CRC [35]. *C11orf53* is a potential gene involved in CRC etiology [36]. *CHI3L1* promotes macrophage recruitment and angiogenesis in CRC [37]. Lastly, alterations of *CDH24* contribute to tumorigenesis, as *CDH24* is important to the maintenance of cell adhesion [38].

Another nine genes of the COAD signature have known associations with other types of cancers, but not with CRC yet. *DNALI1* [39] and *MEOX1* [40] are associated with breast cancer. In particular, *MEOX1* is correlated with poor survival of breast cancer patients. *MARCH11* has been used as a biomarker in a methylation panel for early cancer detection and prognosis prediction in non-small cell lung cancer [41]. *ARHGAP15* is correlated with survival in early-stage pancreatic ductal adenocarcinoma [42]. *IGHA1* is associated with gastric tumorigenesis [43]. *CER1* is associated with glioma [44]. *SDR9C7* promotes lymph node metastasis in esophageal squamous cell carcinoma [45]. *PRKG2* is associated with acute mast cell leukemia [46]. Finally, *ZNF133* is potential biomarker for osteosarcoma [47].

## Discussion

Cancer genomic data is increasingly becoming a hot topic in precision cancer medicine research, including the identification of therapeutic targets, biomarker-based clinical trials, and the study of genomic determinants of therapy response [48]. The signatures found in the present retrospective study are promising and their potential clinical integration should be further investigated with a prospective study.

While the results are promising, there are limitations to this initial work. Demographic and clinical data were not incorporated into the prognostic models. Gene expression data is also available for TCGA LUAD and COAD datasets. Integration of all data types could potentially improve prognostic and risk stratification performance and provide further biological insights. Furthermore, all types of cancer in TCGA should be analyzed for a future pan-cancer study.

The present study was also done at the gene level. There is potential that specific mutations to a gene may have different prognostic effects. However, with the sample size of TCGA data, it is not feasible to observe statistically significant results due to the increased sparsity of somatic mutation data at the specific mutation level. Further data or methods to mitigate the increased sparsity is required for further study.

The present work demonstrated the robustness of prognostic signatures using MFR profiles within TCGA LUAD and COAD VarScan-based somatic mutation data [49] by the partitioning of the data into training and validation datasets. As a result, the experimental and analysis protocols are consistent. The robustness with respect to different somatic mutation calling software within TCGA should be conducted, as calls from MuSE [50], MuTect [51], and SomaticSniper [52] are provided in addition to VarScan. Furthermore, the methods robustness to data generated from different experimental protocols, such as by investigating data generated by different institutions and projects, should be studied in the future.

## Conclusions

To improve clinical tools and biological understanding of LUAD and COAD, we demonstrated a methodology to generating robust prognostic somatic mutation-based gene signatures. We demonstrated the robustness of MFR profiles and its potential to be a powerful prognostic tool in cancer, unlike other alternative types of somatic mutation profiles, TMF, BM, and NetNorM, that did not achieve statistically significant risk stratification in validation datasets. The genes selected by the methodology using MFR profiles was shown to be biologically relevant and has potential for use in effective management LUAD and COAD.

## Methods

### Somatic mutation data and profiles

Controlled TCGA somatic mutation data (VarScan MAF files [49]) were downloaded from NCI’s Genomic Data Commons (https://gdc.cancer.gov/, accessed: Feb. 14, 2018) for LUAD and COAD (Project ID 17109, A Pan-Cancer Analysis of Somatic Mutation Profiles for Tumor Immunogenicity and Prognosis). The data were filtered, keeping only missense mutations. The missense mutations were then aggregated into gene level mutation profiles. For BM profiles, the gene is flagged as mutated if it contains any missense mutation.

The NetNorM normalization method was used as a representative of somatic mutation profiles using gene-gene network information [8]. NetNorM uses networks from Pathway Commons (http://www.pathwaycommons.org), which feature an integrated network data of public pathway and interaction databases. The user-specified parameter for NetNorM is the target number of mutated genes *k*. This parameter is set to the median number of mutated genes in the training dataset, which is 193 and 151 for LUAD and COAD, respectively. NetNorM ranks genes based on their mutation status and network connectedness. A patient’s somatic mutation profile is normalized by setting only the top *k* genes as being mutated. Since mutated genes are always ranked higher than non-mutated genes, patients with more than *k* mutated genes will have lower ranked mutated genes set to unmutated, while patients with less than *k* mutated genes will obtain artificial proxy mutated genes.

### Mutation frequency ratio and tumor frequency profiles

For patient *i*, the MAF files contain the number of reads supporting the reference allele for mutation *j*, *TRC*_*ij*_ and *NRC*_*ij*_ for tumor and normal samples, respectively. Analogously, denote the number of reads supporting the alternate allele, *TAC*_*ij*_ and *NAC*_*ij*_ for tumor and normal samples, respectively. The tumor and normal sample mutation frequencies, *TMF*_*ij*_ and *NMF*_*ij*_, are computed using Eqs. (1) and (2), respectively. The mutation frequency ratio *MFR*_*ij*_ is then simply the ratio of the tumor to normal sample mutation frequencies. To generate a patient’s gene level MFR and TMF profiles, the mutations are aggregated by gene using the mean ratio or frequency within that gene.

1 $$ {TMF}_{ij}=\frac{TAC_{ij}}{TRC_{ij}} $$

2 $$ {NMF}_{ij}=\frac{NAC_{ij}}{NRC_{ij}} $$

3 $$ {MFR}_{ij}=\frac{TMF_{ij}}{NMF_{ij}} $$

### Signature generation and statistical analysis

TCGA clinical data were downloaded from NCI’s Genomic Data Commons (https://gdc.cancer.gov**/**, accessed: Feb. 14, 2018) for LUAD and COAD. These data were partitioned randomly into training (*n* = 328 for LUAD and *n* = 286 for COAD) and validation (*n* = 167 for LUAD and *n* = 141 COAD) datasets. Rarely mutated genes in somatic mutation profiles were omitted when less than 1% of patients in a sample have the mutation. MFR and TMF profiles, which are continuous valued, were *log2* transformed. Univariate Cox proportional hazards regression was used to assess association with overall survival using R survival package (R v3.4.0, survival v2.41–3) with a Benjamini-Hochberg FDR cutoff of 0.05. Multivariate Cox proportional hazards regression was performed using bidirectional stepwise model selection with the R MASS package (MASS v7.3–47). Kaplan-Meier analysis was used to assess risk stratification with R survival and GGally packages (GGally v1.3.2). Pathway and network analysis weres performed with Ingenuity Pathway Analysis.

## Abbreviations

BM: Binary mutation
COAD: Colorectal adenocarcinoma
HR: Hazard ratio
KM: Kaplan-Meier
LUAD: Lung adenocarcinoma
MFR: Mutation frequency ratio
TCGA: The Cancer Genome Atlas
TMF: Tumor mutation frequency
